# Cryptococcal meningitis: A neglected NTD?

**DOI:** 10.1371/journal.pntd.0005575

**Published:** 2017-06-29

**Authors:** Síle F. Molloy, Tom Chiller, Gregory S. Greene, Jessica Burry, Nelesh P. Govender, Cecilia Kanyama, Sayoki Mfinanga, Sokoine Lesikari, Yacouba N. Mapoure, Charles Kouanfack, Victor Sini, Elvis Temfack, David R. Boulware, Francoise Dromer, David W. Denning, Jeremy Day, Neil R. H. Stone, Tihana Bicanic, Joseph N. Jarvis, Olivier Lortholary, Thomas S. Harrison, Shabbar Jaffar, Angela Loyse

**Affiliations:** 1 Centre for Global Health, Institute for Infection and Immunity, St George's, University of London, London, United Kingdom; 2 Mycotic Diseases Branch, Centers for Disease Control and Prevention, Atlanta, Georgia, United States of America; 3 Medecins Sans Frontières Access Campaign, Médecins Sans Frontières, Geneva, Switzerland; 4 National Institute for Communicable Diseases (Centre for Healthcare-Associated Infections, Antimicrobial Resistance and Mycoses), Johannesburg, South Africa; 5 Division of Medical Microbiology, Faculty of Health Sciences, University of Cape Town, Cape Town, South Africa; 6 University of North Carolina Project, Lilongwe, Malawi; 7 National Institute of Medial Research, Muhimbili Medical Research Centre, Dar Es Salaam, Tanzania; 8 Central Hospital, Yaoundé, Cameroon; 9 Department of Internal Medicine, Douala General Hospital, Douala, Cameroon; 10 Division of Infectious Disease and International Medicine, Department of Medicine, University of Minnesota, Minneapolis, Minnesota, United States of America; 11 Institut Pasteur, Molecular Mycology—CNRS URA3012, Department of Mycology, Paris, France; 12 Global Action Fund for Fungal Infections (GAFFI), Geneva, Switzerland; 13 Oxford University Clinical Research Unit, Wellcome Trust Major Overseas Programme Vietnam, Ho Chi Minh City, Vietnam; 14 Centre for Tropical Medicine, Nuffield Department of Medicine, University of Oxford, Oxford, United Kingdom; 15 Department of Clinical Research, Faculty of Infectious & Tropical Diseases, London School of Hygiene and Tropical Medicine, London, United Kingdom; 16 Botswana–University of Pennsylvania Partnership, Gaborone, Botswana; 17 Division of Infectious Diseases, Department of Medicine, Perelman School of Medicine, University of Pennsylvania, Philadelphia, Pennsylvania, United States of America; 18 Department of International Public Health, Liverpool School of Tropical Medicine, Liverpool, United Kingdom; University of Washington, UNITED STATES

Although HIV/AIDS has been anything but neglected over the last decade, opportunistic infections (OIs) are increasingly overlooked as large-scale donors shift their focus from acute care to prevention and earlier antiretroviral treatment (ART) initiation. Of these OIs, cryptococcal meningitis, a deadly invasive fungal infection, continues to affect hundreds of thousands of HIV patients with advanced disease each year and is responsible for an estimated 15%–20% of all AIDS-related deaths [1, 2]. Yet cryptococcal meningitis ranks amongst the most poorly funded “neglected” diseases in the world, receiving 0.2% of available relevant research and development (R&D) funding, according to Policy Cures’ 2016 Global Funding of Innovation for Neglected Diseases (G-Finder) Report [3, 4].

Although cryptococcal meningitis is not formally recognised by the World Health Organisation (WHO) or *PLOS Neglected Tropical Diseases* (*PLOS NTDs*) as a neglected tropical disease (NTD), it is listed in the G-Finder report, as it disproportionately affects people in low- and middle-income countries (LMICs), with market failure evident for existing essential antifungal medicines and an urgent need for new, effective, and less toxic medicines. *PLOS NTDs* defines NTDs as a “group of poverty-promoting chronic infectious diseases, which primarily occur in rural areas and poor urban areas of LMICs” [5] and according to the WHO, NTDs are “a proxy for poverty and disadvantage”, have “an important impact on morbidity and mortality”, and are relatively “neglected by research” [6]. Although the greatest burden of cryptococcal disease is undoubtedly related to HIV, we demonstrate herein that cryptococcal meningitis meets both the WHO and *PLOS NTDs* definitions of an NTD, as the disease (1) disproportionately affects populations in poverty and causes substantial morbidity and mortality, (2) primarily affects populations living in tropical and subtropical areas, (3) is immediately amenable to broad control, elimination, or eradication, and (4) is neglected by research [7].

## Morbidity, mortality, and poverty associated with cryptococcal meningitis

As the most common cause of meningoencephalitis in sub-Saharan Africa and Southeast Asia, cryptococcal meningitis disproportionately affects populations in some of the most economically disadvantaged regions of the world [1–3]. Mortality rates remain high, ranging between 20% and 60% with treatment and up to 100% without, affecting the most economically productive age groups [1, 8–11]. Significant morbidity is often encountered, with severe headaches occurring weeks to months before presentation and potential long-term sequelae including blindness and deafness [12–14]. Such morbidity and mortality places a substantial economic burden on patients and their families, resulting in lost productivity and wages as well as patient-transportation and medical costs. Effective treatments are intensive, expensive (for example, where available, first-line induction therapy with amphotericin B therapy costing over US$200 in Uganda), and available only in hospitals (due to the need for intravenous [IV] administration of amphotericin B and need for close laboratory monitoring for toxicities) [15, 16].

## A disease of the tropics and subtropics

ART scale-up is ultimately the key to reducing cryptococcal meningitis mortality, as has been demonstrated in Europe and North America [17, 18]. However, the burden of morbidity and mortality in LMICs has remained largely unchanged and shows no sign of abating [2, 19–22]. The number of severely immunosuppressed patients not yet accessing ART has remained relatively constant and, in some instances, even increased [23–28]. Data from a recent meta-analysis estimated that over half of patients in Africa had progressed to AIDS with CD4 counts <200 cells/mm^3^ by the time they initiated ART [29], despite over a decade of ART program implementation and scale-up.

While new WHO guidance on a strategy of universal HIV testing and treatment has the potential to prevent cryptococcal disease, poor retention in care and ART failure currently have a significant impact on the incidence of cryptococcal meningitis in Africa. In many hospitals, over half of cryptococcal meningitis cases now occur in patients previously prescribed ART, either in the early months following initiation of ART as unmasking of subclinical infection or, more commonly, later, as a consequence of nonadherence and/or ART failure [28, 30]. There is no sign of a reduction in the high mortality rates for HIV-associated cryptococcal meningitis, driven by late presentation, delayed diagnosis, and inadequate access to and efficacy of current antifungal medicines [31]. Though a number of HIV programs in South and Southeast Asia have achieved some success in terms of ART retention and early detection, the proportion of patients presenting to care with blood samples testing positive for cryptococcal antigen (CrAg) rival those seen in many settings in Africa, demonstrating that cryptococcal meningitis still presents a significant problem, even if absolute numbers of cases are lower [32–35].

## Lack of R&D

A common misconception is that funding for cryptococcal meningitis comes under the umbrella of HIV. In reality, cryptococcal meningitis has fallen into a research and policy gap [4]. The research gap is most evident in drug development, as only 3 antifungal medicines—amphotericin B, flucytosine (5-FC), and fluconazole—are currently used for the treatment of cryptococcal meningitis, all of which are decades old [21, 31]. Current WHO guidelines recommend 2 weeks of amphotericin B and 5-FC as the initial intensive induction phase, followed by a step-down to fluconazole for the consolidation and maintenance phases of treatment [36, 37]. However, this gold standard induction phase remains aspirational for most LMICs. Both amphotericin B and 5-FC are unavailable in the majority of LMICs, and the cheaper, more widely available fluconazole is an inadequate treatment for induction therapy [10]. In addition to patchy drug registration, amphotericin B is often not administered due to issues with toxicity (i.e., anaemia and renal impairment), poor laboratory monitoring facilities, cost barriers, and requirements for cold-chain shipment/storage at 4°C [31, 38]. Although there are 3 stringent regulatory authority (SRA)-approved generic manufacturers of 5-FC (Meda Pharm [recently acquired by Mylan], Sigmapharm, Valeant), none are currently registered in Africa [31, 38, 39, 40] despite compelling data for 5-FC’s use in combination with either amphotericin B or fluconazole during the induction phase of treatment (regimens both recommended in the latest WHO guidelines [8, 14, 36, 37, 40]). Instead, fluconazole is widely available, well tolerated, and used for all phases of cryptococcal treatment despite clear evidence that fluconazole alone is a poorly effective medicine when used during the intensive induction phase, with 10-week mortality consistently >50% [8, 9, 10, 41].

R&D into the manufacture of new or improved therapies is currently inadequate. In 2015, R&D for cryptococcal meningitis totalled US$5.8 million (just over 1% of the R&D budget afforded to tuberculosis [US$567 million]), placing it in the bottom tier of neglected diseases, receiving support essentially from 3 public health funders: United States National Institutes of Health (NIH), United Kingdom Medical Research Council (MRC), and Australian National Health and Medical Research Council [3]. Funding for HIV/AIDS does not currently encompass OIs such as cryptococcal meningitis and, as yet, neither has it fallen under the umbrella of NTD funding. Very few new drugs for cryptococcal meningitis are in the human safety and dose-finding trial stage of development. VT-1129 and VT-1598 (Viamet Pharmaceuticals) are long-acting, azole-like compounds developed with assistance from the NIH’s Therapeutics for Rare and Neglected Diseases programme. In vitro and animal models have demonstrated excellent activity and good oral pharmacokinetics, safety profile, and central nervous system penetration (www.viamet.com/pipeline.asp [42]). In addition, T-2307, which targets the fungal mitochondrial membrane, has completed initial human safety and dose-finding trials in the US (www.toyama-chemical.co.jp/en/rd/pipeline/index.html [43]). Oral formulations of amphotericin B (Matinas Biopharma) and compounds with activity against *Cryptococcus neoformans* (including AN 2690 [Anacor Pharmaceuticals], ASP 2397 [VICAL], and Ar 12 [Arno Therapeutics]) are currently only in preclinical stages of development. No additional new antifungal medicines have been developed for the treatment of cryptococcal meningitis in over 2 decades.

## Strategies for control of cryptococcal meningitis

Despite the challenges, 2 strategies for the control and treatment of cryptococcal meningitis exist that together could make a significant impact: screen-and-treat programs for CrAg and delivery of short-course improved therapies [6]. CrAg screening of severely immunosuppressed patients (typically with CD4 counts ≤100 cells/mm^3^), which is recommended by the WHO and the President's Emergency Plan for AIDS Relief (PEPFAR), combined with preemptive antifungal treatment can prevent or detect at an early stage a significant proportion of cryptococcal meningitis cases, as the antigen can be detected in blood for weeks to months prior to the development of clinical disease. There are sensitive and specific CrAg lateral flow assays (CrAg LFAs) that can be used for diagnosis either in the laboratory or at the point of care. The strategy is highly cost-effective and has recently shown a mortality benefit when combined with increased adherence support and early detection of tuberculosis (TB) [16, 44]. Although CrAg screening has been adopted into policy by over 20 countries, few have committed resources to implementing such programs; attention brought to cryptococcal meningitis as an NTD could help move such policy to action and begin saving lives.

With regard to treatment, optimized, sustainable regimens using existing antifungal medicines, drug development, and testing of repurposed drugs could significantly reduce the case fatality rate. Simplified treatment regimens consisting of short-course combination amphotericin B therapy and a purely oral combination of fluconazole and 5-FC are currently being tested in African LMICs with results expected in 2017 (ACTA trial, ISRCTN45035509). Liposomal formulations of amphotericin B offer a far better-tolerated alternative to traditional formulations. The AMBITION-cm trial is comparing the efficacy of 1 high dose of liposomal amphotericin in combination with fluconazole for HIV-associated cryptococcal meningitis (AMBITION-cm, ISRCTN10248064). A few intermittent doses could prove cost-effective if hospital admission duration was reduced, and there is precedent for efforts to increase access. In September 2016, Gilead extended a donation program to the WHO to make an additional 380,000 vials of liposomal amphotericin available at cost to continue treating patients with visceral leishmaniasis over 5 years [45]. If Gilead’s program were expanded to cover cryptococcal meningitis, this could make improved, simplified treatment of meningitis far more accessible to resource-limited populations where disease burden is highest.

Repurposing of established drugs has also been applied to the search for new medicines [46]. This approach may expedite translation into clinical practice and reduce drug-development costs. Sertraline and tamoxifen are 2 such drugs that are fungicidal against *C*. *neoformans*, with good central nervous system penetration and synergy with fluconazole [30]. Both drugs are being studied separately as new adjunctive therapies for HIV-associated meningitis.

Lastly, raised intracranial pressure affects approximately one third of patients, and managing this common, debilitating, life-threatening complication improves patient outcomes [47, 48]. However, access to manometers, used to measure intracranial pressure, remains inadequate in LMICs and needs to be addressed alongside validation of simple measures to determine flow rates of cerebrospinal fluid (CSF) through spinal needles [49].

## Consideration for inclusion in NTD list

Whilst debate continues as to whether cryptococcal meningitis should be listed as an NTD, affected persons in LMICs remain neglected, with opportunities missed to both prevent disease and provide effective treatment to reduce mortality. Stating that increased ART coverage alone will obviate the problem of HIV-associated cryptococcal meningitis in LMICs is not tenable; the evidence, with striking data from Botswana of ongoing cryptococcal meningitis cases despite excellent ART coverage, shows that this has not been the case to date [22]. Patients with advanced HIV-related cryptococcal meningitis will continue to present to hospitals in LMICs for the foreseeable future.

In 2013, the Cryptococcal Meningitis Action Group (CryptoMAG) was formed to improve access to diagnostic tests and essential antifungal medicines and to disseminate clinical best practices for treatment and prevention in LMICs. The debate over whether or not cryptococcal disease is an NTD detracts from cryptococcal meningitis being both HIV related and also urgently needing the interventions (funding, policy drives, and drug pipelines) from which NTDs benefit. We therefore call on the global health community, *PLOS NTDs*, UNITAID, The Global Fund, and WHO to declare cryptococcal meningitis an NTD and press for urgent funding and policy drives to target optimisation and rollout of CrAg-screening programs, access to 5-FC and liposomal amphotericin B, and new drug development.

## References

[1] Park BJ, Wannemuehler KA, Marston BJ, Govender N, Pappas PG, Chiller TM. Estimation of the current global burden of cryptococcal meningitis among persons living with HIV/AIDS. AIDS. 2009; 23(4):525–30. doi: 10.1097/QAD.0b013e328322ffac 19182676 10.1097/QAD.0b013e328322ffac

[2] Rajasingham R, Smith RM, Park BJ, Jarvis JN, Govender NP, Chiller TM, Denning DW, Loyse A, Boulware DR. Global Burden of Disease of HIV-Associated Cryptococcal Meningitis: an Updated Analysis. Lancet ID. 2017; S1473-3099(17):30243–8. 10.1016/S1473-3099(17)30243-8 PMC5818156 28483415

[3] Chapman N, Abela-Oversteegen L, Doubell A, Chowdhary V, Gurjav, U, Ong M. Neglected disease research and development: A pivotal moment for global health. Policy Cures Research. G-Finder Report 2016. Available from: http://www.policycuresresearch.org/downloads/Y9%20GFINDER%20full%20report%20web.pdf. Accessed: 2016 Sep 14.

[4] Rodrigues ML. Funding and Innovation in Diseases of Neglected Populations: The Paradox of Cryptococcal Meningitis. PLoS Negl Trop Dis. 2016;10(3): e0004429 doi: 10.1371/journal.pntd.0004429 26964103 10.1371/journal.pntd.0004429 PMC4786104

[5] PLoS NTD journal information [Internet]. Available from: http://journals.plos.org/plosntds/s/journal-information. Accessed: 2016 Sep 14.

[6] World Health Organisation. Sustaining the drive to overcome the global impact of neglected tropical diseases: third WHO report on neglected tropical diseases World Health Organization. WHO Press, Geneva, Switzerland, 2015.

[7] WHO NTD application. The WHO Strategic and technical advisory group for neglected tropical diseases (WHO STAG). Recommendations for the adoption of additional diseases as neglected tropical diseases. World Health Organization, Geneva, Switzerland Available from: http://www.who.int/neglected_diseases/diseases/Adoption_additional_NTDs.pdf. Accessed: 2016 Sep 14.

[8] Nussbaum JC, Jackson A, Namarika D, Phulusa J, Kenala J, Kanyemba, et al Combination flucytosine and high dose fluconazole is superior to fluconazole monotherapy for cryptococcal meningitis: a randomized trial in Malawi. Clin Infect Dis. 2010; 50:338–44. doi: 10.1086/649861 20038244 10.1086/649861 PMC2805957

[9] Schaars CF, Meintjes G, Morroni C, Post FA, Maartens G. Outcome of AIDS-associated cryptococcal meningitis initially treated with 200 mg/day or 400 mg/day of fluconazole. BMC Infect Dis. 2006; 6:118 doi: 10.1186/1471-2334-6-118 16846523 10.1186/1471-2334-6-118 PMC1540428

[10] Longley N, Muzoora C, Taseera K, Mwesigye J, Rwebembera J, Chakera A, et al Dose response effect of high-dose fluconazole for HIV-associated cryptococcal meningitis in southwestern Uganda. Clin Infect Dis. 2008; 47(12):1556–61. doi: 10.1086/593194 18990067 10.1086/593194

[11] Bicanic T, Jarvis JN, Loyse A, Jackson A, Muzoora C, Wilson D, et al Determinants of Acute Outcome and Long-term Survival in HIV-associated Cryptococcal Meningitis: Results from a Combined Cohort of 523 Patients. Conf Retroviruses Opportunistic Infect. 2011; 58 (5): 736–45.

[12] Carlson RD, Rolfes MA, Birkenkamp KE, Nakasujja N, Rajasingham R, Meya DB, et al Predictors of neurocognitive outcomes on antiretroviral therapy after cryptococcal meningitis: A prospective cohort study. Metab Brain Dis. 2014; 29(2):269–79. doi: 10.1007/s11011-013-9476-1 24399496 10.1007/s11011-013-9476-1 PMC4033836

[13] Rothe C, Sloan DJ, Goodson P, Chikafa J, Mukaka M, Denis B, et al A prospective longitudinal study of the clinical outcomes from cryptococcal meningitis following treatment induction with 800 mg oral fluconazole in Blantyre, Malawi. PLoS ONE. 2013; 28;8(6). 10.1371/journal.pone.0067311 PMC3696104 23840659

[14] Day JN, Chau TH, Wolbers M, Mai P.P, Dung NT, Mai NH, et al Combination antifungal therapy for cryptococcal meningitis. NEJM. 2013; 368(14):1291–302. doi: 10.1056/NEJMoa1110404 23550668 10.1056/NEJMoa1110404 PMC3978204

[15] Meya D, Rajasingham R, Nalintya E, Tenforde M, Jarvis JN. Preventing Cryptococcosis-Shifting the Paradigm in the Era of Highly Active Antiretroviral Therapy. Curr Trop Med reports. 2015; 2(2):81–9. 10.1007/s40475-015-0045-z PMC4412515 25960942

[16] Jarvis JN, Harrison TS, Lawn SD, Meintjes G, Wood R, Cleary S, et al Cost effectiveness of cryptococcal antigen screening as a strategy to prevent HIV-associated cryptococcal meningitis in South Africa. PLoS ONE. 2013; 8(7): e69288 doi: 10.1371/journal.pone.0069288 23894442 10.1371/journal.pone.0069288 PMC3716603

[17] Mirza SA, Phelan M, Rimland D, Graviss E, Hamill R, Brandt ME, et al The changing epidemiology of cryptococcosis: An update from population-based active surveillance in 2 large metropolitan areas, 1992–2000. Clin Infect Dis. 2003; 36(6):1992–2000. 10.1086/368091 12627365

[18] Dromer F, Mathoulin-Pélissier S, Fontanet A, Ronin O, Dupont B, Lortholary O. Epidemiology of HIV-associated cryptococcosis in France (1985–2001): comparison of the pre- and post-HAART eras. AIDS. 2004; 18(3):555–62. 15090810 10.1097/00002030-200402200-00024

[19] Wall EC, Everett D.B, Mukaka M, Bar-Zeev N, Feasey N, Jahn A, et al Bacterial meningitis in malawian adults, adolescents, and children during the era of antiretroviral scale-up and haemophilus influenzae type B vaccination, 2000–2012. Clin Infect Dis. 2014; 58 (10): e137–e145. doi: 10.1093/cid/ciu057 24496208 10.1093/cid/ciu057 PMC4001285

[20] Jarvis JN, Boulle A, Loyse A, Bicanic T, Rebe K, Williams A, et al High ongoing burden of cryptococcal disease in Africa despite antiretroviral roll out. AIDS. 2009;23(9):1182–3. doi: 10.1097/QAD.0b013e32832be0fc 19451796 10.1097/QAD.0b013e32832be0fc PMC2871312

[21] Govender NP, Chiller TM, Poonsamy B, Frean JA. Neglected fungal diseases in sub-Saharan Africa: A call to action. Curr Fungal Infect Rep. 2011;5 (4):224–32.

[22] Tenforde M, Mokomane M, Leeme T, et al. HIV-associated cryptoccoccal meningitis in Botswana: national incidence and temporal trends following ART rollout. Poster Present. AIDS conf 2016, Durban, South Africa. Available from: http://programme.aids2016.org/Abstract/Abstract/7745. Accessed: 2016 Sep 15.

[23] Boulle A, Bock P, Osler M, Cohen K, Channing L, Hilderbrand K, et al Antiretroviral therapy and early mortality in South Africa. Bull World Heal Organ. 2008; 86:678–87. 10.2471/BLT.07.045294 PMC2649489 18797643

[24] World Health Organisation. HIV in the WHO African Region: progress towards achieving universal access to priority health sector interventions: 2011 update World Health Organisation (WHO) Regional Office for Africa 2011

[25] Lawn SD, Harries AD, Anglaret X, Myer L, Wood R. Early mortality among adults accessing antiretroviral treatment programmes in sub-Saharan Africa. AIDS. 2008; 22(15):1897–908. doi: 10.1097/QAD.0b013e32830007cd 18784453 10.1097/QAD.0b013e32830007cd PMC3816249

[26] Lahuerta M, Wu Y, Hoffman S, Elul B, Kulkarni SG, Remien RH, et al Advanced HIV disease at entry into HIV Care and Initiation of Antiretroviral Therapy during 2006–2011: Findings from four sub-saharan African countries. Clin Infect Dis. 2014; 58(3):432–41. doi: 10.1093/cid/cit724 24198226 10.1093/cid/cit724 PMC3890338

[27] IeDEA and ART Cohort Collaborations, Avila D, Althoff KN, Mugglin C, Wools-Kaloustian K, Koller M, et al Immunodeficiency at the start of combination antiretroviral therapy in low-, middle-, and high-income countries. J Acquir Immune Defic Syndr. 2014; 65(1):e8–16. doi: 10.1097/QAI.0b013e3182a39979 24419071 10.1097/QAI.0b013e3182a39979 PMC3894575

[28] Jarvis JN, Meintjes G, Harrison TS. Outcomes of cryptococcal meningitis in antiretroviral naive and experienced patients in South Africa. J Infect. 2010; 60(6):496–8. doi: 10.1016/j.jinf.2010.03.007 20307571 10.1016/j.jinf.2010.03.007 PMC2879388

[29] Siedner MJ, Ng CK, Bassett I V, Katz IT, Bangsberg DR, Tsai AC. Trends in CD4 Count at Presentation to Care and Treatment Initiation in Sub-Saharan Africa, 2002–2013: A Meta-analysis. Clin Infect Dis. 2015; 60(7):1120–7. doi: 10.1093/cid/ciu1137 25516189 10.1093/cid/ciu1137 PMC4366582

[30] Rhein J, Morawski BM, Hullsiek KH Nabeta HW, Kiggundu R, Tugume L, et al Efficacy of adjunctive sertraline for the treatment of HIV-associated cryptococcal meningitis: An open-label dose-ranging study. Lancet Infect Dis 2016; 16 (7)809–818. doi: 10.1016/S1473-3099(16)00074-8 26971081 10.1016/S1473-3099(16)00074-8 PMC4927382

[31] Loyse A, Thangaraj H, Easterbrook P, Ford N, Roy M, Chiller TM, et al Cryptococcal meningitis: improving access to essential antifungal medicines in resource poor countries. Lancet Infect Dis. 2013; 13(7): 629–637. doi: 10.1016/S1473-3099(13)70078-1 23735626 10.1016/S1473-3099(13)70078-1

[32] Matsumoto S, Tanuma J, Mizushima D, Nguyen NCT, Pham TTT, Do CD, et al High treatment retention rate in HIV-infected patients receiving antiretroviral therapy at two large HIV clinics in Hanoi, Vietnam. PLoS ONE. 2015; 10(9). 10.1371/journal.pone.0139594 PMC4589350 26422474

[33] Vun MC, Fujita M, Rathavy T, Eang MT, Sopheap S, Sovannarith S, et al Achieving universal access and moving towards elimination of new HIV infections in Cambodia. J Int AIDS Soc 2014; 17 17:18905. 24950749 10.7448/IAS.17.1.18905 PMC4065309

[34] Ganiem AR, Indrati AR, Wisaksana R, Meijerink H, Van Der Ven A, Alisjahbana B, et al Asymptomatic cryptococcal anigenaemia is associated with mortality among HIV positive in Indonesia. J Int AIDS Soc 2014; 17:18821 doi: 10.7448/IAS.17.1.18821 24476751 10.7448/IAS.17.1.18821 PMC3906483

[35] Kwan CK, Leelawiwat W, Intalapaporn P, Anekthananon T, Raengsakulrach B, Peters PJ, et al Utility of cryptococcal antigen screening and evolution of asymptomatic cryptococcal antigenemia among HIV-infected women starting antiretroviral therapy in Thailand. J Int Assoc Physicians AIDS Care. 2014; 13(5):434–7. 10.1177/2325957413500533 24003059

[36] Perfect JR, Dromer F, Dismukes WE, Goldman DL, Graybil JR, Hamill RJ, et al Clinical Practice Guidelines for the Management of Cryptococcal Disease: 2010 Update by the Infectious Diseases Society of America. Clin Infect Dis. 2010; 50(3):291–322. doi: 10.1086/649858 20047480 10.1086/649858 PMC5826644

[37] World Health Organisation. Consolidated guidelines on the use of antiretroviral drugs for treating and preventing HIV infection WHO Press, Geneva, Switzerland, 6, 2013. 24716260

[38] Kneale M, Bartholomew JS, Davies E, Denning DW. Global access to antifungal therapy and its variable cost. J Antimicrob Chemother. 2016; 71(12):3599–3606. doi: 10.1093/jac/dkw325 27516477 10.1093/jac/dkw325

[39] Jarvis JN, Boulle A, Loyse A, Bicanic T, Rebe K, Williams A, et al High ongoing burden of cryptococcal disease in Africa despite antiretroviral roll out. AIDS. 2009; 23(9):1182–3 doi: 10.1097/QAD.0b013e32832be0fc 19451796 10.1097/QAD.0b013e32832be0fc PMC2871312

[40] Loyse A, Dromer F, Day J, Lortholary O & Harrison TS. Flucytosine and cryptococcosis: time to urgently address the worldwide accessibility of a 50 year old antifungal. Journal of Antimicrobial Chemotherapy 2013; 68(11): 2435–2444. doi: 10.1093/jac/dkt221 23788479 10.1093/jac/dkt221 PMC3797641

[41] Bicanic T, Harrison TS, Niepieklo A, Dyakopu N, Meintjes G. Symptomatic relapse of HIV-associated cryptococcal meningitis after initial fluconazole monotherapy: the role of fluconazole resistance and immune reconstitution. Clin Infect Dis. 2006; 43(8):1069–73. doi: 10.1086/507895 16983622 10.1086/507895

[42] Lockhart SR, Fothergill AW, Iqbal N, Bolden CB, Grossman NT, Garvey EP, et al The investigational fungal Cyp51 Inhibitor VT-1129 demonstrates potent *in vitro* activity against Cryptococcus neoformans and Cryptococcus gattii. Antimicrob Agents Chemother. 2016; 60(4):2528–31. doi: 10.1128/AAC.02770-15 26787697 10.1128/AAC.02770-15 PMC4808209

[43] Shibata T, Takahashi T, Yamada E, Kimura A, Nishikawa H, Hayakawa H, Nomura N, Mitsuyama J. T-2307 causes collapse of mitochondrial membrane potential in yeast. Antimicrob. Agents Chemother. 2012; 56:5892–5897. doi: 10.1128/AAC.05954-11 22948882 10.1128/AAC.05954-11 PMC3486560

[44] Mfinanga S, Chanda D, Kivuyo SL, Guinness L, Bottomley C, Simms V, et al Cryptococcal meningitis screening and community-based early adherence support reduces all-cause mortality among HIV-infected people initiating antiretroviral therapy with advanced disease: a randomised-controlled trial in Tanzania and Zambia. Lancet. 2015; 385:2173–82. doi: 10.1016/S0140-6736(15)60164-7 25765698 10.1016/S0140-6736(15)60164-7

[45] Gilead. Visceral Leishmaniasis Treatment Expansion. Available from: http://www.gilead.com/responsibility/developing-world-access/visceral%20leishmaniasis. Accessed: 2016 Sep 15.

[46] Krysan DJ. Toward improved anti-cryptococcal drugs: novel molecules and repurposed drugs. Fungal Genet Biol. 2015; 78:93–8. doi: 10.1016/j.fgb.2014.12.001 25514636 10.1016/j.fgb.2014.12.001

[47] Rolfes MA, Hullsiek KH, Rhein J, Nabeta HW, Taseera K, Schutz C, et al The Effect of Therapeutic Lumbar Punctures on Acute Mortality from Cryptococcal Meningitis. Clin Infect Dis. 2014; 59(11):1607–14. doi: 10.1093/cid/ciu596 25057102 10.1093/cid/ciu596 PMC4441057

[48] Bicanic T, Brouwer AE, Meintjes G, Rebe K, Limmathurotsakul D, Chierakul W, et al Relationship of cerebrospinal fluid pressure, fungal burden and outcome in patients with cryptococcal meningitis undergoing serial lumbar punctures. AIDS 2009;23(6):701–6. doi: 10.1097/QAD.0b013e32832605fe 19279443 10.1097/QAD.0b013e32832605fe

[49] Boyles TH, Gately E, Wasserman S, Meintjes G. Flow rate of CSF through a spinal needle can accurately predict intracranial pressure in cryptococcal meningitis. J Acquir Immune Defic Syndr. 2016; 74(3): e64–e66. 10.1097/QAI.0000000000001183 28187086

